# Integrating artificial with natural cells to translate chemical messages that direct *E. coli* behaviour

**DOI:** 10.1038/ncomms5012

**Published:** 2014-05-30

**Authors:** Roberta Lentini, Silvia Perez Santero, Fabio Chizzolini, Dario Cecchi, Jason Fontana, Marta Marchioretto, Cristina Del Bianco, Jessica L. Terrell, Amy C. Spencer, Laura Martini, Michele Forlin, Michael Assfalg, Mauro Dalla Serra, William E. Bentley, Sheref S. Mansy

**Affiliations:** 1 CIBIO, University of Trento, via delle Regole 101, 38123 Mattarello (TN), Italy; 2 Department of Biotechnology, University of Verona, 37134 Verona, Italy; 3 National Research Council—Institute of Biophysics & Bruno Kessler Foundation, Via alla Cascata 56/C, 38123 Trento, Italy; 4 Fischell Department of Bioengineering, University of Maryland, College Park, Maryland 20742, USA; 5 Institute for Bioscience and Biotechnology Research, University of Maryland, College Park, Maryland 20742, USA

## Abstract

Previous efforts to control cellular behaviour have largely relied upon various forms of genetic engineering. Once the genetic content of a living cell is modified, the behaviour of that cell typically changes as well. However, other methods of cellular control are possible. All cells sense and respond to their environment. Therefore, artificial, non-living cellular mimics could be engineered to activate or repress already existing natural sensory pathways of living cells through chemical communication. Here we describe the construction of such a system. The artificial cells expand the senses of *Escherichia coli* by translating a chemical message that *E. coli* cannot sense on its own to a molecule that activates a natural cellular response. This methodology could open new opportunities in engineering cellular behaviour without exploiting genetically modified organisms.

## Abstract (web summary)

The control of cellular behaviour largely relies on genetic engineering, but artificial cells could be designed to control cell processes through chemical communication. Here, the authors develop an artificial cell that is able to translate a chemical message into a signal that can be sensed by *E. coli* and activate a cellular response.

## Graphical abstract

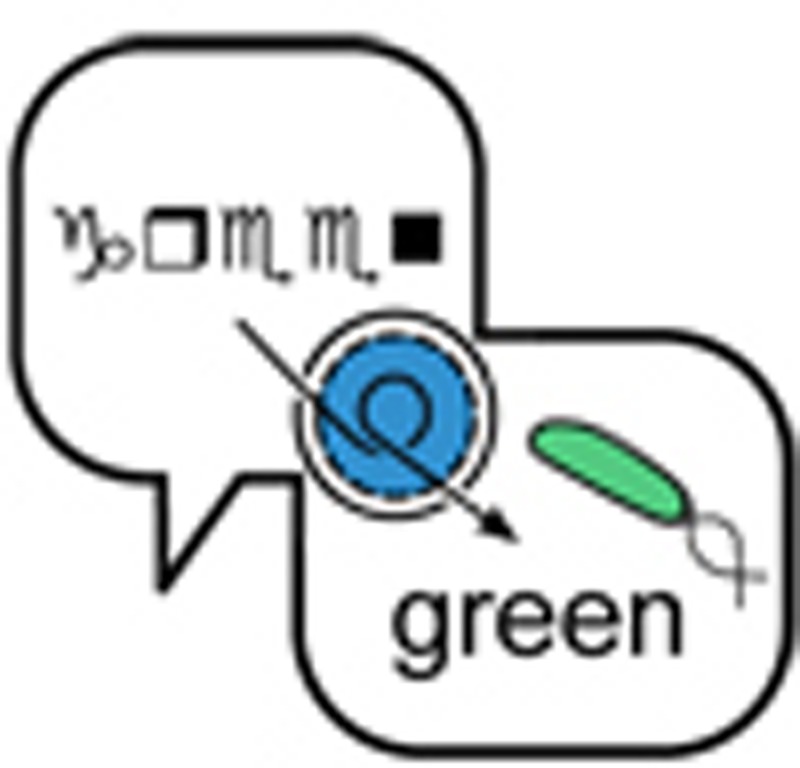

Synthetic biology thus far has relied upon the engineering of new cellular function through the insertion and deletion of genetic information in living cells. This genetic engineering based approach has progressed rapidly. There is now available a set of well-characterized biological parts^1^,^2^,^3^ that can be used to build complex genetic circuitry within and between the living cells^4^,^5^,^6^. Further, entire genomes can be edited^7^ and synthesized^8^, suggesting that fully designed organisms with heretofore unseen capabilities are likely in the future.

Despite the wide range of technologies and target pathways exploited, the desire to control microorganisms to date has always employed direct genetic intervention. The limitations of these prevalent methods are due to the difficulties of engineering living systems, including evolutionary pressures that may alter engineered pathways over time and the potential long-term consequences of altering ecosystems with engineered organisms. However, it may not be necessary to genetically modify living cells. Extant life is already extremely complex, endowed with numerous sensory and metabolic pathways tuned by billions of years of evolution to be efficiently responsive to changing intracellular and extracellular conditions. A simple change in pH, for example, results in the up and downregulation of nearly 1,000 genes in *Escherichia coli*^9^. In other words, cells are already capable of sensing many different stimuli and capable of performing many tasks. Therefore, it should be possible to exploit these existing cellular pathways to control cellular behaviour without changing the genetic makeup of the cells.

Here we explore this idea of engineering *E. coli* through alternative means by targeting the sensory pathways of *E. coli*. To do so without altering the genetic content of the bacterium, we instead construct artificial cells that could interact with natural cells in order to evoke a behavioural response. The artificial cells in this system function as chemical translators that sense molecules that *E. coli* alone cannot sense. In response, the artificial cells release a molecule that *E. coli* can naturally respond to, thereby translating an unrecognized chemical message into a recognized chemical message. In this way, the sensory capabilities of *E. coli* are expanded without altering the genetic content of the bacterium. The artificial cell is built with a phospholipid vesicle containing isopropyl β-D-1-thiogalactopyranoside (IPTG), DNA, and transcription–translation machinery. The DNA template codes for a previously selected riboswitch that activates translation in response to the presence of theophylline^10^. The theophylline riboswitch controls the synthesis of the pore forming protein α-hemolysin (αHL). Therefore, in the presence, but not the absence, of theophylline a pore forms that releases entrapped IPTG. *E. coli* alone does not respond to theophylline, and IPTG does not cross the vesicle membrane of the artificial cell in the absence of the pore. The ability of *E. coli* to receive the chemical message sent by the artificial cells is assessed in two ways. First, the fluorescence of *E. coli* carrying a plasmid encoding a fluorescent protein behind an IPTG-responsive, *lac* operator sequence is evaluated. Second, the gene expression of untransformed *E. coli* is monitored by reverse transcription quantitative PCR (RT–qPCR). To our knowledge, this is the first artificial, cell-like system capable of translating unrecognized signals into a chemical language that natural cells can recognize. The integration of artificial translator cells with natural cells represents a new strategy to introduce synthetic features to a biological system while circumventing the need for direct genetic manipulation.

## Results

### The theophylline-sensing device is functional *in vitro*

**Figure 1:**
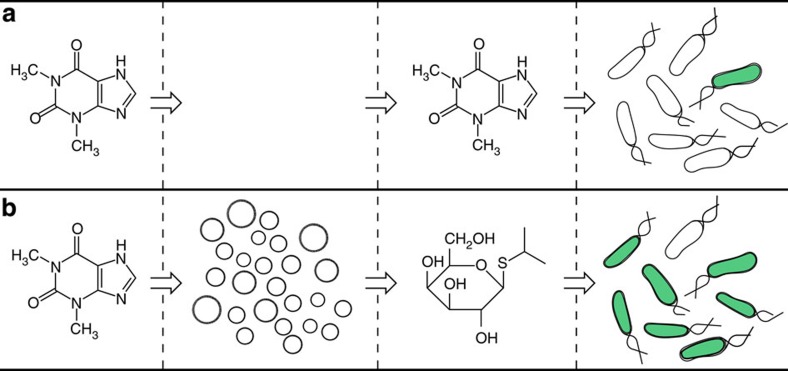
Artificial cells translate chemical signals for *E. coli*. (**a**) In the absence of artificial cells (circles), *E. coli* (oblong) cannot sense theophylline. (**b**) Artificial cells can be engineered to detect theophylline and in response release IPTG, a chemical signal that induces a response in *E. coli*.

**Figure 2:**
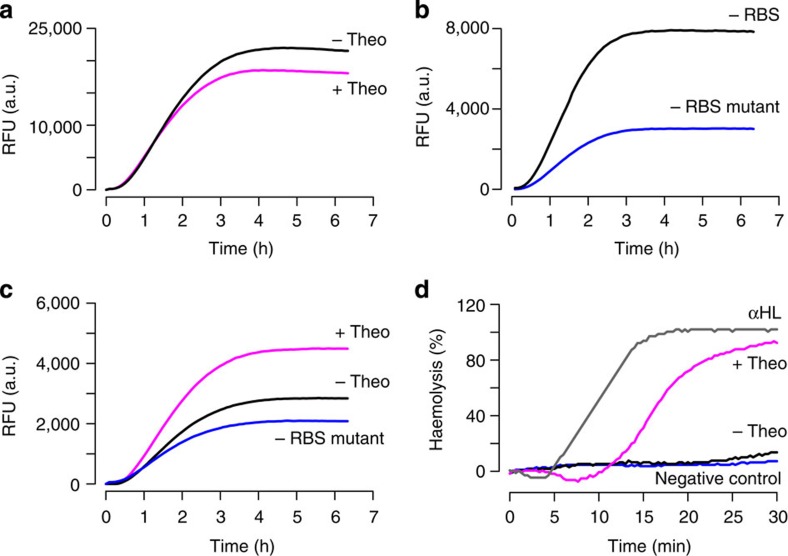
*In vitro* characterization of the theophylline-sensing device and αHL. (**a**) The cell-free expression of αHL–GFP behind a theophylline riboswitch gives rise to similar levels of fluorescence both in the presence (+theo) and absence (−theo) of theophylline at 37 °C. (**b**) The removal of the theophylline riboswitch and thus, the RBS preceding the start codon of αHL–GFP shows production of a fluorescent protein product when incubated with transcription–translation machinery (−RBS). The removal of a putative internal RBS within the αHL coding portion of the fusion construct significantly decreases the production of the fluorescent protein product (−RBS mutant). (**c**) The activity of the theophylline-sensing device is observable by fluorescence when an internal RBS is removed. The top and middle curves are the *in vitro* expression of αHL–GFP behind the theophylline riboswitch in the presence (+theo) and absence of theophylline (−theo), respectively. Background fluorescent protein production is shown with the same construct lacking the theophylline riboswitch (−RBS mutant) used in **b**. (**d**) The cell-free expression of theophylline riboswitch-controlled αHL-degraded red blood cells (RBCs) in the presence (+theo) but not the absence of theophylline (−theo). Control reactions include the expression of an αHL construct lacking the theophylline riboswitch (αHL) and RBCs alone (negative control). RBC degradation was monitored by attenuance at 22 °C. The exploited constructs were SP011A for panel A, SP002A and AS014A for panel B, RL069A and AS014A for **c**, and RL067A and JF001A for **d** (Supplementary Table 1). Data are averages of three independent reactions.

To build artificial cells that sense theophylline and in response release IPTG (Fig. 1), a theophylline-sensing genetic device was built with a T7 transcriptional promoter, a theophylline riboswitch and a gene encoding a fusion between αHL and super folder GFP at the carboxy terminus. If functioning properly, this arrangement should result in the expression of protein and thus green fluorescence only in the presence of theophylline. However, cell-free expression in the presence and absence of theophylline showed similar levels of fluorescence (Fig. 2a). Since this same riboswitch was previously shown to function *in vitro*^11^, the sequence of the αHL-GFP gene was more closely examined. Multiple pairs of potential ribosome binding sites (RBS) and start codons were identified within the αHL portion of the gene that were in-frame with the GFP-encoding region. The theophylline riboswitch controls translation, meaning that sequences behind the theophylline riboswitch are always transcribed. Translation from the RBS within the riboswitch is activated by direct binding of theophylline to the messenger RNA. Therefore, if additional sequences outside of the riboswitch but within the αHL portion of the gene were recognized by the ribosome, then regardless of the theophylline concentration, the expression of truncated peptide products with fluorescently active GFP would have been possible. To test if such internal RBSs were present, the theophylline riboswitch and thus the RBS preceding the αHL-GFP sequence was deleted. *In vitro* transcription–translation of this construct showed the accumulation of fluorescence over time similar to the riboswitch containing construct (Fig. 2b). Sequence analysis revealed three potential RBS-start codon pairs within the αHL coding portion of the gene. Of these, a putative RBS of AAAGAA was selected as the most likely candidate for giving fluorescent protein expression based on sequence composition and spacing^12^. The putative internal RBS was removed by mutation to TCTACC, resulting in a carboxy-terminal GFP tagged K30S E31T αHL construct. Fluorescence from this mutated construct was reduced threefold, consistent with the removal of an internal RBS (Fig. 2b). Finally, K30S E31T αHL-GFP was placed behind the theophylline riboswitch to test the activity of the cell-free sensing device. A clear difference was observed between protein expression in the presence and absence of theophylline (Fig. 2c), and the fluorescence arising in the absence of theophylline was within 20% of the construct lacking an RBS upstream of the full gene. The data were consistent with a functioning riboswitch sensor with background fluorescent protein expression arising from internal RBS within αHL. Therefore, the final artificial cellular mimic described below was built with αHL lacking a GFP-tag to avoid complications arising from the expression of truncated fluorescent protein product.

### Active αHL is produced in response to theophylline *in vitro*

To ensure that the cell-free expressed αHL was active as a pore, the ability of αHL to degrade rabbit red blood cells was assessed through a standard haemolysis assay^13^. Each construct was expressed *in vitro* at 37 °C for 6 h after which, an aliquot was removed and added to red blood cells. Haemolysis was quantified by measuring attenuance at 650 nm. In the presence of theophylline, 90% haemolysis was observed when the genetic construct containing a riboswitch-controlled αHL was expressed. The cell-free expression of the same construct in the absence of theophylline gave haemolysis levels similar to the negative control reactions (Fig. 2d), as was expected for a functioning theophylline riboswitch that controls the production of αHL. Control reactions with commercial αHL-purified protein and *in vitro*-expressed αHL and αHL–GFP all were fully active (Fig. 2d, Supplementary Table 2), whereas aliquots from *in vitro*-expressed GFP alone and αHL with a carboxy-terminal His-tag were inactive (Supplementary Table 2). αHL with a carboxy-terminal His-tag was previously shown to have reduced activity^14^. Also, comparison of the riboswitch activity fluorescence data with the haemolysis assay data was consistent with the production of GFP containing protein fragments from an internal RBS without an active αHL domain. For example, the αHL–GFP construct lacking one of the putative internal RBSs failed to produce protein with haemolysis activity (Supplementary Table 2), despite giving rise to fluorescence during *in vitro* transcription–translation (Fig. 2b).

### Artificial cells can translate chemical messages for *E. coli*

After demonstrating that the riboswitch was able to control the *in vitro* expression of αHL in response to theophylline and that the expressed αHL molecules formed functional pores, the component parts were next assembled inside of phospholipid vesicles to build artificial cells. Theophylline is capable of passing through the membrane of vesicles^11^. Phospholipid vesicles were generated in the presence of IPTG, transcription–translation machinery and DNA encoding αHL under the control of the theophylline riboswitch. The vesicles were then purified by dialysis at 4 °C to remove unencapsulated molecules. The receiver bacterial cells were mid-exponential phase *E. coli* BL21(DE3) pLysS carrying a plasmid encoding GFP behind a T7 promoter and a *lac* operator sequence. In this commonly exploited system, IPTG induces the expression of a chromosomal copy of T7 RNA polymerase in *E. coli* BL21(DE3) and derepresses the expression of GFP from the plasmid. Background expression is typically low with such an arrangement because of the presence of constitutively expressed lysozyme from pLysS, a natural inhibitor of T7 RNA polymerase.

**Figure 3:**
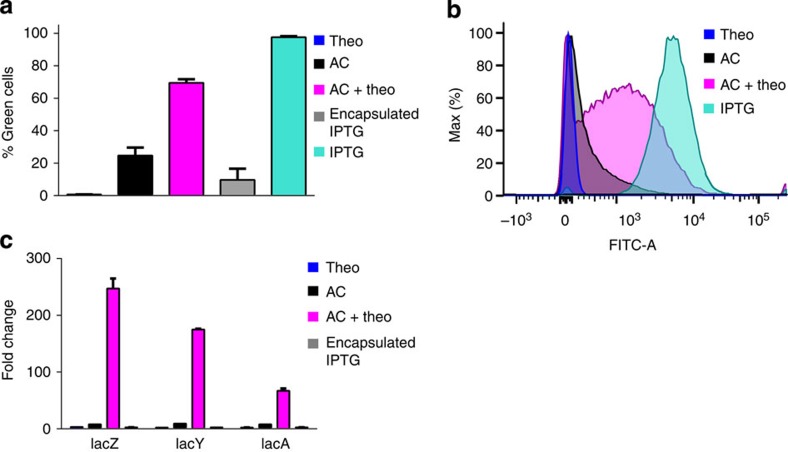
The artificial translator cells are functional. (**a**) Artificial cells can induce the expression of a plasmid encoded gene within *E. coli* in response to a molecule that *E. coli* cannot naturally sense. BL21(DE3) pLysS carrying a plasmid encoding GFP behind a *lac* operator sequence was incubated with the following components at 37 °C for 3 h: theophylline (theo), artificial cells (AC), artificial cells plus theophylline (AC+theo), IPTG encapsulated inside of vesicles (encapsulated IPTG), and unencapsulated IPTG (IPTG). *E. coli* fluorescence was quantified by flow cytometry. The reported averages and s.e.m. were calculated from three separate reactions run on three different days from independently assembled artificial cells. (**b**) A histogram of a subset of the FACS data used in panel **a** shows a clear shift in the *E. coli* population in the presence of artificial cells plus theophylline. (**c**) Artificial cells can induce the expression of chromosomally encoded genes of *E. coli*. After 4 h of incubation of artificial cells with *E. coli* at 37 °C, the messenger RNA encoding *lacZ*, *lacY* and *lacA* was quantified by RT–qPCR. Data are reported as averages of three measurements and error bars represent s.e.m.

To test if the artificial cells could function as chemical translators for *E. coli*, the artificial cells were incubated with *E. coli* BL21(DE3) pLysS carrying the GFP-encoding plasmid at 37 °C, and the fluorescence of *E. coli* was evaluated by flow cytometry. A control reaction in which theophylline was directly added to *E. coli* in the absence of artificial cells failed to show green fluorescence after 3 h (Fig. 3a). Similarly, IPTG loaded vesicles that did not contain the machinery necessary to form pores did not induce fluorescence in *E. coli*. Therefore, theophylline was not able to induce a detectable response in *E. coli*, and IPTG could not cross the vesicle membrane in the absence of αHL, which was consistent with permeability measurements (Supplementary Fig. 1). However, when *E. coli* was incubated with artificial cells and theophylline, 17±10% and 69±3% of the bacteria fluoresced green after 0.5 and 3 h, respectively. When the same experiment was repeated in the absence of theophylline, 3±1% and 24±5% of the bacteria were fluorescent after 0.5 and 3 h, respectively (Fig. 3a,b). Longer incubations resulted in diminishing differences between the two samples suggesting the presence of low levels of αHL expression in the absence of theophylline. Also, the GFP response was encoded within a medium copy number plasmid. Therefore, higher background levels of GFP were to be expected in comparison with gene expression from the chromosome. The flow cytometry experiments were consistent with the ability of artificial cells to translate an unrecognized chemical signal (theophylline) into a signal (IPTG) that *E. coli* could respond to.

Although the artificial cells were capable of communicating with *E. coli*, the induction of GFP synthesis, as observed above, exploited an engineered response. To assess whether artificial cells could elicit a natural, chromosomally encoded response, RT–qPCR was used to measure gene expression from the *lac* operon of *E. coli*. The *lac* operon is one of the most thoroughly characterized sensory pathways^15^. The presence of allolactose (or the non-hydrolyzable analogue IPTG) induces the expression of *lacZ*, *lacY* and *lacA*. To facilitate detection of *E. coli* responding to the chemical message sent from the artificial cells, *E. coli* BL21 (DE3) pLysS were grown in LB supplemented with glucose to decrease the background expression of the *lac* operon and then transferred to M9 minimal media prior to incubation with artificial cells. The artificial cells were prepared as described for the GFP induction experiments above. After incubating together artificial cells with *E. coli* in the presence and absence of theophylline for 4 h, aliquots were collected for RNA isolation. The RNA was then reverse transcribed and *lacZ*, *lacY*, and *lacA* expression quantified by qPCR. The RNA isolated from bacteria incubated with artificial cells plus theophylline showed on average over 20-fold higher *lacZYA* expression than samples incubated with artificial cells alone (calculated from AC/(AC+theo) as shown in Fig. 3c). Taken together, the data are consistent with the ability of artificial cells to translate chemical messages and induce both engineered and natural pathways in *E. coli*.

## Discussion

Direct genetic engineering of living cells is not needed to control cellular behaviour. It is possible, instead, to coerce desired activity through communication with artificial cells. The foundation for such technologies has already been laid by both cell-free and *in vivo* studies. Engineered communication paths between living cells have been constructed to coordinate cellular activities in response to external stimuli^6^,^16^ and are being developed for therapeutic purposes^17^. In these systems, sender cells often can process information and in response release molecules that affect other cells. What has been shown herein builds on these past efforts but does so by integrating reconstituted, non-living systems with living cells. This allows for the genetic engineering component of the system to be moved from the living, evolving, replicating cells to the more controllable, ephemeral artificial cells. When the artificial cells degrade, the natural cells go back to their original state, thereby diminishing the possibility of unintended long-term consequences. For example, rather than engineering bacteria to search for and clean up environmental contaminants, artificial cells could be built to sense the contaminant molecules and in response release chemoattractants that bring natural bacteria capable of feeding on the contaminants^18^ to the affected site.

Several recent reports have described the engineering of seek-and-destroy bacteria for the eradication of tumours or bacterial infections^19^,^20^,^21^,^22^. However, these methods ultimately rely on administering living bacteria to the patient. Artificial cells could be built to carry out similar tasks if the sensor module of the artificial cell was designed to detect the chemical conditions associated with the ailment. For instance, rather than spraying engineered bacteria into the lungs of cystic fibrosis patients, artificial cells could be built to detect the presence of *Pseudomonas aeruginosa* biofilms through the quorum signalling molecules that are naturally secreted by the organism, such as *N*-(3-oxododecanoyl)-L-homoserine lactone, a molecule capable of crossing membranes without the aid of transporters. Subsequently, the artificial cells could release small molecules, for example, D-amino acids^23^, to disperse the biofilm and thus clear the infection. Moreover, the use of dispersion rather than killing would decrease the probability of the bacteria developing resistance. Similar strategies with artificial cells could be developed to substitute for engineered probiotics that integrate with gut microbiota^24^ and prevent disease^25^,^26^.

There are several limitations to these first generation artificial cells. First, heterogeneity in membrane lamellarity and in encapsulation efficiency^27^ results in a mixture of artificial cells with varying degrees of activity. Microfluidic-based methods for compartment formation and solute encapsulation would likely alleviate many of the complications associated with vesicle-to-vesicle and batch-to-batch variability. Also, a system fully dependent upon the permeability properties of the membrane limits the types of molecules that can be sensed and released. The development of specific membrane-associated sensors and transporters will likely be necessary as the complexity of artificial cells increase. Finally, the simple release of encapsulated molecules means that release could result from compartment degradation as opposed to an engineered response to the detection of a specific molecule. It is, therefore, important to develop an output that is mediated by synthesis so that compartment degradation would only result in the release of inactive starting molecules. An example of such a system is the biological nanofactory described by Fernandes *et al.*^28^ that synthesizes a signalling molecule from *S*-(5′-deoxyadenosin-5′)-L-homocysteine via two enzymatic steps.

The absence of a living chassis opens up greater opportunities to assemble or biofabricate various mechanisms or functions that would be difficult to implement with living cells. For example, chemical systems housed within inorganic and peptide-based compartments are capable of sensing the environment through, in part, the gating behaviour of the non-lipid compartment^29^,^30^. Further, artificial cells can synthesize and release signalling molecules sensed by living cells without exploiting genetically encoded parts^31^,^32^. The possibility of merging advances with non-genetically encoded and genetically encoded parts may lead to the construction of artificial cells that are better able to imitate natural cellular life^33^,^34^.

## Methods

### Genetic constructs

The gene encoding *Staphylococcus aureus* αHL was synthesized by Genscript. Super folder GFP (BBa_I746916) was from the registry of standard biological parts ( http://parts.igem.org). The theophylline riboswitch sequence was from Lynch and Gallivan^10^ and was amplified from a previously described construct^11^. All genes were subcloned into pET21b (Novagen) with NdeI and XhoI restriction sites. Mutagenesis was performed by Phusion site-directed mutagenesis (Thermo Scientific). All constructs were confirmed by sequencing at Genechron or Eurofins MWG Operon. Sequences of all the exploited constructs are listed in Supplementary Table 1. All experiments were repeated at least three times. Data are reported as averages with standard error, or representative runs are shown.

### *In vitro* characterization of the riboswitch

Plasmids were amplified in *E. coli* Novablue (Novagen) and purified with Wizard Plus SV Minipreps DNA Purification System (Promega). Plasmid DNA was phenol–chloroform extracted, ethanol precipitated and resuspended in deionized and diethyl pyrocarbonate-treated water. PCR products were purified with Wizard Plus SV Gel and PCR Clean-Up Systems (Promega). Transcription–translation reactions used the PURExpress *In Vitro* Protein Synthesis Kit (New England Biolabs) supplemented with 20 units of Human Placenta RNase Inhibitor (New England Biolabs). Reactions were monitored by fluorescence with a CFX96 Touch real-time PCR (Bio-Rad) using the SYBR green filter set.

### α-hemolysin activity

Each construct was expressed with the PURExpress *In Vitro* Protein Synthesis Kit at 37 °C in a final volume of 25 μl either in the presence or absence of 1.5 mM theophylline for 6 h. Rabbit red blood cell (RBC) suspensions (adjusted to *D*=0.1 at 650 nm) were added to a microplate where the reaction mixtures were serially diluted. Changes in attenuance of the RBC suspension were measured at 650 nm with a microplate reader (UVmax, Molecular Devices) for 30 min at 22 °C as reported in Laventie *et al.*^35^ The results are reported as percentage of haemolysis or as the time necessary to reach 50% of haemolysis.

### Preparation of *E. coli* receiver cells

Mid-exponential *E. coli* BL21(DE3) pLysS transformed with a plasmid encoding super folder GFP behind a T7 promoter and a *lac* operator sequence (CD101A^12^) were grown in LB supplemented with 100 μg ml^−1^ ampicillin and 34 μg ml^−1^ chloramphenicol to an optical density of 0.5 at 600 nm. A quantity of 200 μl aliquots in 10% (vol/vol) glycerol were flash frozen with liquid nitrogen and stored at −80 °C for later use. Aliquots were rapidly thawed and mixed with 2 ml LB supplemented with 100 μg ml^−1^ ampicillin and 34 μg ml^−1^ chloramphenicol and incubated for 2 h at 37 °C with 220 r.p.m. shaking. Finally, the cells were gently pelleted and resuspended in 1 ml M9 minimal media.

### Preparation of artificial cells

Vesicles were prepared as previously described^36^,^37^. Briefly, 12.5 mg 1-palmitoyl-2-oleoyl-*sn*-glycero-3-phosphocholine (POPC) and 12.5 mg cholesterol (Avanti Polar Lipids) in chloroform were mixed in a round bottom flask. A thin lipid film was made through rotary evaporation with a Buchi Rotovapor R-210 equipped with a Buchi Vacuum Pump V-700 for 5 h. A quantity of 2 ml DEPC-treated deionized water was then added to the thin lipid film and vigorously vortexed. The resulting liposome dispersion was homogenized with an IKA T10 basic homogenizer at a power setting of 4 for 1 min. A quantity of 100 μl aliquots were frozen in liquid nitrogen or dry ice and lyophilized overnight in a vacuum concentrator (Centritrap DNA concentrator, Labconco) at 40 °C. The lyophilized empty liposomes were stored at −20 °C. A quantity of 100 μl aliquots of freeze-dried liposomes were hydrated with 25 μl of 100 mM IPTG (Sigma) dissolved in 50 mM HEPES pH7.6, 25 μl of the PURE system, 500 ng DNA and 20 units of human placenta RNase inhibitor (final volume of 50 μl), unless otherwise noted. Solutions were gently mixed for 30 s.

To remove extravesicular material, the vesicles were dialyzed following a method previously described by Zhu and Szostak^38^. The original membranes of 500 μl Slide-a-Lyzer dialysis cassettes (Pierce) were exchanged with 25 mm diameter polycarbonate track-etched membranes with a 1 μm pore size (Whatman). A quantity of 50 μl of unpurified vesicles were loaded onto the center of the dialysis system with a 100 μl Hamilton syringe and dialyzed against 250 ml of buffer A (50 mM HEPES, 10 mM MgCl_2_, 100 mM KCl, pH 7.6) with stirring. The first four rounds of dialysis were for 10 min each. Two more rounds of dialysis in which the buffer was changed after 30 min incubations were further performed. All dialysis steps were carried out at 4 °C.

### Artificial–natural cell communication

Purified vesicles containing DNA, the PURE system, and IPTG were incubated with *E. coli* BL21(DE3) pLysS transformed with CD101A in M9 minimal media supplemented with 1 mg ml^−1^ of Proteinase K and 5 mM theophylline at 37 °C in a final volume of 40 μl. Control reactions did not contain theophylline. At different time points, 1 μl was removed and diluted 1:100 in PBS. The sample was then analysed by flow cytometry with a FACSCanto A (BD Biosciences). The FITC filter was used for the detection of positive cells. The incident light was at 488 nm for forward scatter (FSC), side scatter (SSC) and fluorescence. Detection for SSC and fluorescence was at 488±10 nm and 530±30 nm, respectively. The threshold parameters were 200 for both FSC and SSC. The PMT voltage settings were 525 (FSC), 403 (SSC) and 600 (FITC). The flow rate was set to ‘low’. For each sample 30,000 events were collected. Reactions were repeated three times on three separate days. Data were analysed using FlowJo software (TreeStar, USA).

Samples were also evaluated by RT–qPCR. Here, the dialyzed vesicles and *E. coli* were incubated as described above for 4 h at 37 °C. Subsequently, the total RNA was extracted with the RNeasy Mini kit (Qiagen). A quantity of 10 μl of 500 ng of RNA was reverse transcribed using RevertAid Reverse Transcriptase (Thermo Scientific). cDNA was quantified with a CFX96 Touch real-time PCR (Bio-Rad) with SYBR green detection. Each sample was diluted to 5 ng and measured in triplicate in a 96 wells plate (Bio-Rad) in a reaction mixture containing SsoAdvanced SYBR green supermix (Bio-Rad) and 180 nM of each primer in a 10 μl finale volume. The primers used to quantify *lacZ*, *lacY* and *lacA* expression were lacZ FW: 5′-TACGATGCGCCCATCTACAC-3′, lacZ REV: 5′-AACAACCCGTCGGATTCTCC-3′, lacY FW: 5′-GGTTTCCAGGGCGCTTATCT-3′, lacY REV: 5′-TTCATTCACCTGACGACGCA-3′, lacA FW: 5′-GCGTCACCATCGGGGATAAT-3′, lacA REV: 5′-CCACGACGTTTGGTGGAATG-3′. Gene expression was normalized to the expression of *idnT*^39^ with the following primers: 5′-CTGCCGTTGCGCTGTTTATT-3′ and 5′-GATTTGCTCGATGGTGCGTC-3′.

## Author contributions

Design, cloning and mutagenesis of genetic constructs were done by R.L., A.C.S., J.F., S.P.S., M.F., and C.D.B. *In vitro* riboswitch activity was investigated by R.L., S.P.S., C.D.B., L.M., M.F. and A.C.S. αHL activity was measured by R.L., S.P.S., M.M., and M.D.S. R.L., J.L.T., D.C., F.C. and S.P.S. ran the cell flow cytometry experiments, and RT–qPCR was performed by R.L. and J.F. S.S.M. supervised the project. All authors analysed and interpreted the data and contributed to the writing of the manuscript.

## Additional information

**How to cite this article**: Lentini, R. *et al.* Integrating artificial with natural cells to translate chemical messages that direct *E*. coli behaviour. *Nat. Commun.* 5:4012 doi: 10.1038/ncomms5012 (2014).

## Supplementary Material

Supplementary Information Supplementary Figure 1, Supplementary Tables 1-2 and Supplementary References
