# Supplementary material for: Integrating artificial with natural cells to translate chemical messages that direct E. coli behaviour
Source: Nat Commun. 2014 May 30;5:4012. doi: 10.1038/ncomms5012 (PMC4050265; doi:10.1038/ncomms5012)
Supplement: Supplementary Information — Supplementary Figure 1, Supplementary Tables 1-2 and Supplementary References [file ncomms5012-s1.pdf]

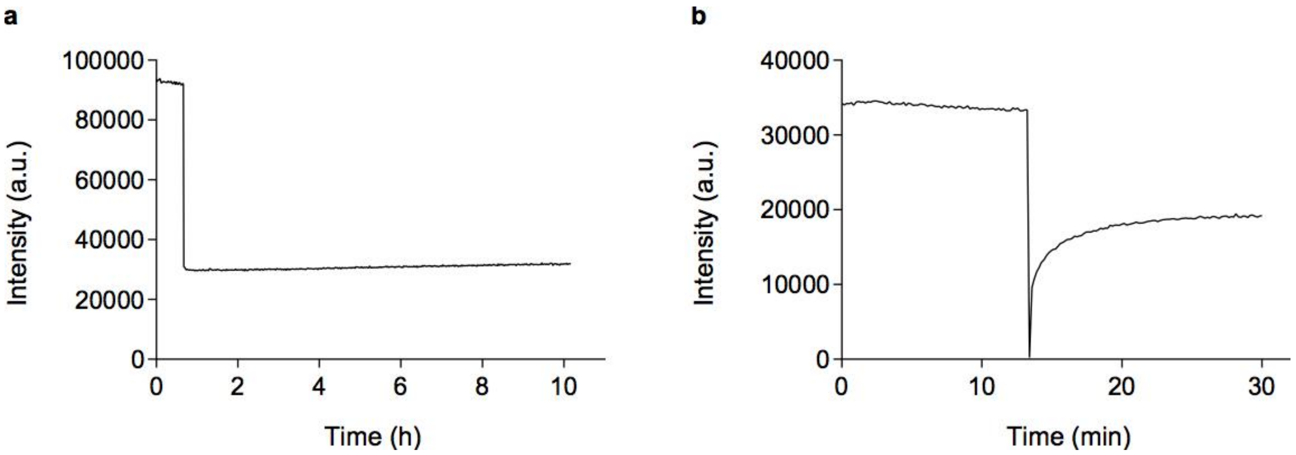

Supplementary Figure 1. Vesicle permeability to IPTG. (A) A shrink-swell[2-4] assay was used to assess whether IPTG was capable of crossing vesicle membranes. POPC:cholesterol vesicles with entrapped calcein were prepared in 10 mM $MgCl_2$, 100 mM KCl, 50 mM HEPES, pH 7.6 as described in the methods and subsequently purified by gel filtration chromatography with sepharose-4b. An aliquot of the vesicle sample was diluted two-fold with 1.0 M IPTG (final concentration = 0.5 M) at 37 °C. The reaction was monitored by fluorescence with excitation and emission wavelengths of 495 nm and 515 nm, respectively. The rapid decrease in fluorescence was due to both dilution with the solute solution and calcein self-quenching. If IPTG were capable of crossing the membrane, a recovery of fluorescence would have been observed. (B) The permeability of POPC vesicles to ribose was observable with the shrink-swell assay. The recovery in fluorescence after two-fold dilution with 1.0 M ribose (final concentration = 0.5 M) was due to equilibration of ribose and water across the membrane.

Supplementary Table 1. DNA sequences used in this study

NAME	NOTE	SEQUENCE
AS014A	T7 promoter, K30S E31T αHL-sfGFP	ATTTAATACGACTCACTATAG**ATG**GATTCTGATATCAATATCAAAACCGGCACCACCGATATCGGCTC<br>CAATACCACCGTTAAAACCGGTGATCTGGTGACCTATGATTCTACCAACGGTATGCATAAAAAAGTGT<br>TTTACTCGTTTATTGACGATAAAAACCATAACAAAAAACTGCTGGTCATCCGCACCAAAGGCACCATTG<br>CGGGTCAATACCGTGTGTACTCCGAAGAAGGTGCGAACAAAAGCGGTCTGGCTTGGCCGTCTGCCTT<br>TAAAGTGCAGCTGCAACTGCCGGATAATGAAGTGGCGCAGATTTCAGATTATTATCCGCGTAATAGCA<br>TCGATACCAAAGAATATATGAGTACCCTGACCTATGGTTTTAATGGCAATGTTACCGGTGATGATACG<br>GGTAAAATTGGCGGTCTGATTGGCGCCAATGTGTCCATTGGTCATACGCTGAAATACGTGCAACCGG<br>ATTTCAAAACCATTCTGGAAAGTCCGACCGATAAAAAAGTGGGTTGGAAAGTTATCTTCAACAACATG<br>GTGAATCAGAACTGGGGTCCGTACGATCGCGATTCCTGGAATCCGGTTTATGGCAATCAGCTGTTTAT<br>GAAAACCCGCAACGGTAGTATGAAAGCGGCGGATAATTTTCTGGACCCGAACAAAGCCTCAAGCCTG<br>CTGTCCAGCGGTTTTAGCCCGGATTTTGCCACGGTTATTACCATGGATCGCAAAGCCAGCAAACAGCA<br>GACCAACATTGATGTGATCTACGAACGTGTGCGTGATGATTATCAACTGCATTGGACCTCAACCAATT<br>GGAAAGGCACCAATACCAAAGATAAATGGACGGATCGCAGTTCAGAACGCTACAAAATTGATTGGGA<br>AAAAGAAGAAATGACCAACGGATCCGGCAGCGGTTCT**ATG**CGTAAAGGCGAAGAGCTGTTCACTGG<br>TGTCGTCCCTATTCTGGTGGAACTGGATGGTGATGTCAACGGTCATAAGTTTTCCGTGCGTGGCGAGG<br>GTGAAGGTGACGCAACTAATGGTAAACTGACGCTGAAGTTCATCTGTACTACTGGTAAACTGCCGGTA<br>CCTTGGCCGACTCTGGTAACGACGCTGACTTATGGTGTTCAGTGCTTTGCTCGTTATCCGGACCATATG<br>AAGCAGCATGACTTCTTCAAGTCCGCCATGCCGGAAGGCTATGTGCAGGAACGCACGATTTCCTTTAA<br>GGATGACGGCACGTACAAAACGCGTGCGGAAGTGAAATTTGAAGGCGATACCCTGGTAAACCGCATT<br>GAGCTGAAAGGCATTGACTTTAAAGAAGACGGCAATATCCTGGGCCATAAGCTGGAATACAATTTTA<br>ACAGCCACAATGTTTACATCACCGCCGATAAACAAAAAAATGGCATTAAAGCGAATTTTAAAATTCGC<br>CACAACGTGGAGGATGGCAGCGTGCAGCTGGCTGATCACTACCAGCAAAACACTCCAATCGGTGATG<br>GTCCTGTTCTGCTGCCAGACAATCACTATCTGAGCACGCAAAGCGTTCTGTCTAAAGATCCGAACGAG<br>AAACGCGATCATATGGTTCTGCTGGAGTTCGTAACCGCAGCGGGCATCACGCATGGTATGGATGAAC<br>TGTACAAA**TAA**CTCGAGCACCACCACCACCACCACTGAGATCCGGCTGCTAACAAAGCCCGAAAGGA<br>AGCTGAGTTGGCTGCTGCCACCGCTGAGCAATAACTAGCATAACCCCTTGGGGCCTCTAAACGGGTCT<br>TGAGGGGTTTTTTG
DT101A	T7 promoter, αHL-His tag	TAATACGACTCACTATAGGGGAATTGTGAGCGGATAACAATTCCCCTCTAGAAATAATTTTGTTTAACT<br>TTAAGAAGGAGATATACAT**ATG**GATTCTGATATCAATATCAAAACCGGCACCACCGATATCGGCTCC<br>AATACCACCGTTAAAACCGGTGATCTGGTGACCTATGATAAAGAAAACGGTATGCATAAAAAAGTGTT<br>TTACTCGTTTATTGACGATAAAAACCATAACAAAAAACTGCTGGTCATCCGCACCAAAGGCACCATTGC<br>GGGTCAATACCGTGTGTACTCCGAAGAAGGTGCGAACAAAAGCGGTCTGGCTTGGCCGTCTGCCTTT<br>AAAGTGCAGCTGCAACTGCCGGATAATGAAGTGGCGCAGATTTCAGATTATTATCCGCGTAATAGCAT<br>CGATACCAAAGAATATATGAGTACCCTGACCTATGGTTTTAATGGCAATGTTACCGGTGATGATACGG<br>GTAAAATTGGCGGTCTGATTGGCGCCAATGTGTCCATTGGTCATACGCTGAAATACGTGCAACCGGAT<br>TTCAAAACCATTCTGGAAAGTCCGACCGATAAAAAAGTGGGTTGGAAAGTTATCTTCAACAACATGGT<br>GAATCAGAACTGGGGTCCGTACGATCGCGATTCCTGGAATCCGGTTTATGGCAATCAGCTGTTTATGA<br>AAACCCGCAACGGTAGTATGAAAGCGGCGGATAATTTTCTGGACCCGAACAAAGCCTCAAGCCTGCT<br>GTCCAGCGGTTTTAGCCCGGATTTTGCCACGGTTATTACCATGGATCGCAAAGCCAGCAAACAGCAGA<br>CCAACATTGATGTGATCTACGAACGTGTGCGTGATGATTATCAACTGCATTGGACCTCAACCAATTGG<br>AAAGGCACCAATACCAAAGATAAATGGACGGATCGCAGTTCAGAACGCTACAAAATTGATTGGGAAA<br>AAGAAGAAATGACCAACCTCGAGCACCACCACCACCACCAC**TGA**GATCCGGCTGCTAACAAAGCCCG<br>AAAGGAAGCTGAGTTGGCTGCTGCCACCGCTGAGCAATAACTAGCATAACCCCTTGGGGCCTCTAAAC<br>GGGTCTTGAGGGGTTTTTTG
JF001A	T7 promoter, theophylline riboswitch αHL	AATTAATACGACTCACTATAGGGTGATACCAGCATCGTCTTGATGCCCTTGGCAGCACCCTGCTAAGG<br>TAACAACAAG**ATG**GATTCTGATATCAATATCAAAACCGGCACCACCGATATCGGCTCCAATACCACCG<br>TTAAAACCGGTGATCTGGTGACCTATGATAAAGAAAACGGTATGCATAAA AAAGTGTTTTACTCGTTT<br>ATTGACGATAAAAACCATAACAAAAAACTGCTGGTCATCCGCACCAAAGGCACCATTGCGGGTCAATA<br>CCGTGTGTACTCCGAAGAAGGTGCGAACAAAAGCGGTCTGGCTTGGCCGTCTGCCTTTAAAGTGCAG<br>CTGCAACTGCCGGATAATGAAGTGGCGCAGATTTCAGATTATTATCCGCGTAATAGCATCGATACCAA

		AGAATATATGAGTACCCTGACCTATGGTTTTAATGGCAATGTTACCGGTGATGATACGGGTAAAATTG<br>GCGGTCTGATTGGCGCCAATGTGTCCATTGGTCATACGCTGAAATACGTGCAACCGGATTTCAAAACC<br>ATTCTGGAAAGTCCGACCGATAAAAAAGTGGGTTGGAAAGTTATCTTCAACAACATGGTGAATCAGA<br>ACTGGGGTCCGTACGATCGCGATTCCTGGAATCCGGTTTATGGCAATCAGCTGTTTATGAAAACCCGC<br>AACGGTAGTATGAAAGCGGCGGATAATTTTCTGGACCCGAACAAAGCCTCAAGCCTGCTGTCCAGCG<br>GTTTTAGCCCGGATTTTGCCACGGTTATTACCATGGATCGCAAAGCCAGCAAACAGCAGACCAACATT<br>GATGTGATCTACGAACGTGTGCGTGATGATTATCAACTGCATTGGACCTCAACCAATTGGAAAGGCAC<br>CAATACCAAAGATAAATGGACGGATCGCAGTTCAGAACGCTACAAAATTGATTGGGAAAAAGAAGAA<br>ATGACCAAC**TAA**CTCGAGCACCACCACCACCACCACTGAGATCCGGCTGCTAACAAAGCCCGAAAGG<br>AAGCTGAGTTGGCTGCTGCCACCGCTGAGCAATAACTAGCATAACCCCTTGGGGCCTCTAAACGGGTC<br>TTGAGGGGTTTTTTG
RL067A	T7 promoter, αHL	TAATACGACTCACTATAGGGGAATTGTGAGCGGATAACAATTCCCCTCTAGAAATAATTTTGTTTAACT<br>TTAAG*AAGGAG*ATATACAT**ATG**GATTCTGATATCAATATCAAAACCGGCACCACCGATATCGGCTCC<br>AATACCACCGTTAAAACCGGTGATCTGGTGACCTATGATAAAGAAAACGGTATGCATAAAAAAGTGTT<br>TTACTCGTTTATTGACGATAAAAACCATAACAAAAAACTGCTGGTCATCCGCACCAAAGGCACCATTGC<br>GGGTCAATACCGTGTGTACTCCGAAGAAGGTGCGAACAAAAGCGGTCTGGCTTGGCCGTCTGCCTTT<br>AAAGTGCAGCTGCAACTGCCGGATAATGAAGTGGCGCAGATTTCAGATTATTATCCGCGTAATAGCAT<br>CGATACCAAAGAATATATGAGTACCCTGACCTATGGTTTTAATGGCAATGTTACCGGTGATGATACGG<br>GTAAAATTGGCGGTCTGATTGGCGCCAATGTGTCCATTGGTCATACGCTGAAATACGTGCAACCGGAT<br>TTCAAAACCATTCTGGAAAGTCCGACCGATAAAAAAGTGGGTTGGAAAGTTATCTTCAACAACATGGT<br>GAATCAGAACTGGGGTCCGTACGATCGCGATTCCTGGAATCCGGTTTATGGCAATCAGCTGTTTATGA<br>AAACCCGCAACGGTAGTATGAAAGCGGCGGATAATTTTCTGGACCCGAACAAAGCCTCAAGCCTGCT<br>GTCCAGCGGTTTTAGCCCGGATTTTGCCACGGTTATTACCATGGATCGCAAAGCCAGCAAACAGCAGA<br>CCAACATTGATGTGATCTACGAACGTGTGCGTGATGATTATCAACTGCATTGGACCTCAACCAATTGG<br>AAAGGCACCAATACCAAAGATAAATGGACGGATCGCAGTTCAGAACGCTACAAAATTGATTGGGAAA<br>AAGAAGAAATGACCAAC**TAA**CTCGAGCACCACCACCACCACCACTGAGATCCGGCTGCTAACAAAGC<br>CCGAAAGGAAGCTGAGTTGGCTGCTGCCACCGCTGAGCAATAACTAGCATAACCCCTTGGGGCCTCT<br>AAACGGGTCTTGAGGGGTTTTTTG
RL069A	T7 promoter,<br>theophylline riboswitch,<br>K30S E31T αHL	AATTAATACGACTCACTATAGGGTGATACCAGCATCGTCTTGATGCCCTTGGCAGCACCCTGCTAAGG<br>TAACAACAAG**ATG**GATTCTGATATCAATATCAAAACCGGCACCACCGATATCGGCTCCAATACCACC<br>GTTAAAACCGGTGATCTGGTGACCTATGATTCTACCAACGGTATGCATAAAAAAGTGTTTTACTCGTTT<br>ATTGACGATAAAAACCATAACAAAAAACTGCTGGTCATCCGCACCAAAGGCACCATTGCGGGTCAATA<br>CCGTGTGTACTCCGAAGAAGGTGCGAACAAAAGCGGTCTGGCTTGGCCGTCTGCCTTTAAAGTGCAG<br>CTGCAACTGCCGGATAATGAAGTGGCGCAGATTTCAGATTATTATCCGCGTAATAGCATCGATACCAA<br>AGAATATATGAGTACCCTGACCTATGGTTTTAATGGCAATGTTACCGGTGATGATACGGGTAAAATTG<br>GCGGTCTGATTGGCGCCAATGTGTCCATTGGTCATACGCTGAAATACGTGCAACCGGATTTCAAAACC<br>ATTCTGGAAAGTCCGACCGATAAAAAAGTGGGTTGGAAAGTTATCTTCAACAACATGGTGAATCAGA<br>ACTGGGGTCCGTACGATCGCGATTCCTGGAATCCGGTTTATGGCAATCAGCTGTTTATGAAAACCCGC<br>AACGGTAGTATGAAAGCGGCGGATAATTTTCTGGACCCGAACAAAGCCTCAAGCCTGCTGTCCAGCG<br>GTTTTAGCCCGGATTTTGCCACGGTTATTACCATGGATCGCAAAGCCAGCAAACAGCAGACCAACATT<br>GATGTGATCTACGAACGTGTGCGTGATGATTATCAACTGCATTGGACCTCAACCAATTGGAAAGGCAC<br>CAATACCAAAGATAAATGGACGGATCGCAGTTCAGAACGCTACAAAATTGATTGGGAAAAAGAAGAA<br>ATGACCAACGGATCCGGCAGCGGTTCT**ATG**CGTAAAGGCGAAGAGCTGTTCACTGGTGTCGTCCCTA<br>TTCTGGTGGAACTGGATGGTGATGTCAACGGTCATAAGTTTTCCGTGCGTGGCGAGGGTGAAGGTGA<br>CGCAACTAATGGTAAACTGACGCTGAAGTTCATCTGTACTACTGGTAAACTGCCGGTACCTTGGCCGA<br>CTCTGGTAACGACGCTGACTTATGGTGTTCAGTGCTTTGCTCGTTATCCGGACCATATGAAGCAGCAT<br>GACTTCTTCAAGTCCGCCATGCCGGAAGGCTATGTGCAGGAACGCACGATTTCCTTTAAGGATGACGG<br>CACGTACAAAACGCGTGCGGAAGTGAAATTTGAAGGCGATACCCTGGTAAACCGCATTGAGCTGAAA<br>GGCATTGACTTTAAAGAAGACGGCAATATCCTGGGCCATAAGCTGGAATACAATTTTAACAGCCACAA<br>TGTTTACATCACCGCCGATAAACAAAAAAATGGCATTAAAGCGAATTTTAAAATTCGCCACAACGTGG<br>AGGATGGCAGCGTGCAGCTGGCTGATCACTACCAGCAAAACACTCCAATCGGTGATGGTCCTGTTCTG<br>CTGCCAGACAATCACTATCTGAGCACGCAAAGCGTTCTGTCTAAAGATCCGAACGAGAAACGCGATCA<br>TATGGTTCTGCTGGAGTTCGTAACCGCAGCGGGCATCACGCATGGTATGGATGAACTGTACAAA**TAA**

		CTCGAGCACCACCACCACCACCACTGAGATCCGGCTGCTAACAAAGCCCGAAAGGAAGCTGAGTTGG<br>CTGCTGCCACCGCTGAGCAATAACTAGCATAACCCCTTGGGGCCTCTAAACGGGTCTTGAGGGGTTTT<br>TTG
SP002A	T7 promoter,<br>αHL-sfGFP	ATTTAATACGACTCACTATAG**ATG**GATTCTGATATCAATATCAAAACCGGCACCACCGATATCGGCTC<br>CAATACCACCGTTAAAACCGGTGATCTGGTGACCTATGATAAAGAAAACGGTATGCATAAAAAAGTGT<br>TTTACTCGTTTATTGACGATAAAAACCATAACAAAAAACTGCTGGTCATCCGCACCAAAGGCACCATTG<br>CGGGTCAATACCGTGTGTACTCCGAAGAAGGTGCGAACAAAAGCGGTCTGGCTTGGCCGTCTGCCTT<br>TAAAGTGCAGCTGCAACTGCCGGATAATGAAGTGGCGCAGATTTCAGATTATTATCCGCGTAATAGCA<br>TCGATACCAAAGAATATATGAGTACCCTGACCTATGGTTTTAATGGCAATGTTACCGGTGATGATACG<br>GGTAAAATTGGCGGTCTGATTGGCGCCAATGTGTCCATTGGTCATACGCTGAAATACGTGCAACCGG<br>ATTTCAAAACCATTCTGGAAAGTCCGACCGATAAAAAAGTGGGTTGGAAAGTTATCTTCAACAACATG<br>GTGAATCAGAACTGGGGTCCGTACGATCGCGATTCCTGGAATCCGGTTTATGGCAATCAGCTGTTTAT<br>GAAAACCCGCAACGGTAGTATGAAAGCGGCGGATAATTTTCTGGACCCGAACAAAGCCTCAAGCCTG<br>CTGTCCAGCGGTTTTAGCCCGGATTTTGCCACGGTTATTACCATGGATCGCAAAGCCAGCAAACAGCA<br>GACCAACATTGATGTGATCTACGAACGTGTGCGTGATGATTATCAACTGCATTGGACCTCAACCAATT<br>GGAAAGGCACCAATACCAAAGATAAATGGACGGATCGCAGTTCAGAACGCTACAAAATTGATTGGGA<br>AAAAGAAGAAATGACCAACGGATCCGGCAGCGGTTCT**ATG**CGTAAAGGCGAAGAGCTGTTCACTGG<br>TGTCGTCCCTATTCTGGTGGAACTGGATGGTGATGTCAACGGTCATAAGTTTTCCGTGCGTGGCGAGG<br>GTGAAGGTGACGCAACTAATGGTAAACTGACGCTGAAGTTCATCTGTACTACTGGTAAACTGCCGGTA<br>CCTTGGCCGACTCTGGTAACGACGCTGACTTATGGTGTTCAGTGCTTTGCTCGTTATCCGGACCATATG<br>AAGCAGCATGACTTCTTCAAGTCCGCCATGCCGGAAGGCTATGTGCAGGAACGCACGATTTCCTTTAA<br>GGATGACGGCACGTACAAAACGCGTGCGGAAGTGAAATTTGAAGGCGATACCCTGGTAAACCGCATT<br>GAGCTGAAAGGCATTGACTTTAAAGAAGACGGCAATATCCTGGGCCATAAGCTGGAATACAATTTTA<br>ACAGCCACAATGTTTACATCACCGCCGATAAACAAAAAAATGGCATTAAAGCGAATTTTAAAATTCGC<br>CACAACGTGGAGGATGGCAGCGTGCAGCTGGCTGATCACTACCAGCAAAACACTCCAATCGGTGATG<br>GTCCTGTTCTGCTGCCAGACAATCACTATCTGAGCACGCAAAGCGTTCTGTCTAAAGATCCGAACGAG<br>AAACGCGATCATATGGTTCTGCTGGAGTTCGTAACCGCAGCGGGCATCACGCATGGTATGGATGAAC<br>TGTACAAA**TAA**CTCGAGCACCACCACCACCACCACTGAGATCCGGCTGCTAACAAAGCCCGAAAGGA<br>AGCTGAGTTGGCTGCTGCCACCGCTGAGCAATAACTAGCATAACCCCTTGGGGCCTCTAAACGGGTCT<br>TGAGGGGTTTTTTG
SP011A	T7 promoter,<br>theophylline riboswitch,<br>αHL-sfGFP	AATTAATACGACTCACTATAGGGTGATACCAGCATCGTCTTGATGCCCTTGGCAGCACCCTGCTAAGG<br>TAACAACAAG**ATG**GATTCTGATATCAATATCAAAACCGGCACCACCGATATCGGCTCCAATACCACC<br>GTTAAAACCGGTGATCTGGTGACCTATGATAAAGAAAACGGTATGCATAAAAAAGTGTTTTACTCGTT<br>TATTGACGATAAAAACCATAACAAAAAACTGCTGGTCATCCGCACCAAAGGCACCATTGCGGGTCAAT<br>ACCGTGTGTACTCCGAAGAAGGTGCGAACAAAAGCGGTCTGGCTTGGCCGTCTGCCTTTAAAGTGCA<br>GCTGCAACTGCCGGATAATGAAGTGGCGCAGATTTCAGATTATTATCCGCGTAATAGCATCGATACCA<br>AAGAATATATGAGTACCCTGACCTATGGTTTTAATGGCAATGTTACCGGTGATGATACGGGTAAAATT<br>GGCGGTCTGATTGGCGCCAATGTGTCCATTGGTCATACGCTGAAATACGTGCAACCGGATTTCAAAAC<br>CATTCTGGAAAGTCCGACCGATAAAAAAGTGGGTTGGAAAGTTATCTTCAACAACATGGTGAATCAG<br>AACTGGGGTCCGTACGATCGCGATTCCTGGAATCCGGTTTATGGCAATCAGCTGTTTATGAAAACCCG<br>CAACGGTAGTATGAAAGCGGCGGATAATTTTCTGGACCCGAACAAAGCCTCAAGCCTGCTGTCCAGC<br>GGTTTTAGCCCGGATTTTGCCACGGTTATTACCATGGATCGCAAAGCCAGCAAACAGCAGACCAACAT<br>TGATGTGATCTACGAACGTGTGCGTGATGATTATCAACTGCATTGGACCTCAACCAATTGGAAAGGCA<br>CCAATACCAAAGATAAATGGACGGATCGCAGTTCAGAACGCTACAAAATTGATTGGGAAAAAGAAGA<br>AATGACCAACGGATCCGGCAGCGGTTCT**ATG**CGTAAAGGCGAAGAGCTGTTCACTGGTGTCGTCCCT<br>ATTCTGGTGGAACTGGATGGTGATGTCAACGGTCATAAGTTTTCCGTGCGTGGCGAGGGTGAAGGTG<br>ACGCAACTAATGGTAAACTGACGCTGAAGTTCATCTGTACTACTGGTAAACTGCCGGTACCTTGGCCG<br>ACTCTGGTAACGACGCTGACTTATGGTGTTCAGTGCTTTGCTCGTTATCCGGACCATATGAAGCAGCAT<br>GACTTCTTCAAGTCCGCCATGCCGGAAGGCTATGTGCAGGAACGCACGATTTCCTTTAAGGATGACGG<br>CACGTACAAAACGCGTGCGGAAGTGAAATTTGAAGGCGATACCCTGGTAAACCGCATTGAGCTGAAA<br>GGCATTGACTTTAAAGAAGACGGCAATATCCTGGGCCATAAGCTGGAATACAATTTTAACAGCCACAA<br>TGTTTACATCACCGCCGATAAACAAAAAAATGGCATTAAAGCGAATTTTAAAATTCGCCACAACGTGG<br>AGGATGGCAGCGTGCAGCTGGCTGATCACTACCAGCAAAACACTCCAATCGGTGATGGTCCTGTTCTG

		CTGCCAGACAATCACTATCTGAGCACGCAAAGCGTTCTGTCTAAAGATCCGAACGAGAAACGCGATCA<br>TATGGTTCTGCTGGAGTTCGTAACCGCAGCGGGCATCACGCATGGTATGGATGAACTGTACAAA**TAA**<br>CTCGAGCACCACCACCACCACCACTGAGATCCGGCTGCTAACAAAGCCCGAAAGGAAGCTGAGTTGG<br>CTGCTGCCACCGCTGAGCAATAACTAGCATAACCCCTTGGGGCCTCTAAACGGGTCTTGAGGGGTTTT<br>TTG

Start and stop codons are in bold and the theophylline riboswitch is underlined. The T7 promoter and T7 terminator sequences are TAATACGACTCACTATA and CTAGCATAACCCCTTGGGGCCTCTAAACGGGTCTTGAGGGGTTTTTTG, respectively.

Supplementary Table 2. The activity of cell-free expressed αHL

construct name	$t_{1/2}$ (min)	Comments
JF001A	> 30	αHL behind theophylline riboswitch in the absence of theophylline
JF001A	16.5	αHL behind theophylline riboswitch in the presence of theophylline
DT101A	> 30	αHL-His tagged
SP002A	4.5	αHL-GFP
RL067A	10.0	αHL
Sigma-Aldrich αHL	9.5	commercial αHL
CD101A[1]	> 30	GFP
AS014A	> 30	K30S E31T αHL-GFP lacking an internal RBS

Each construct was expressed *in vitro* with the PURE system and subsequently added to rabbit red blood cells. Hemolysis was measured by attenuance as described in the methods. When indicated, the theophylline concentration was 1.5 mM. Sigma-Aldrich αHL indicates purchased purified protein and was not *in vitro* expressed.

Supplementary References

1. Lentini R., *et al.* Fluorescent proteins and in vitro genetic organization for cell-free synthetic biology. *ACS Synth. Biol.* **2**, 482-489 (2013).

2. Bittman R. & Blau L. Permeability behavior of liposomes prepared from fatty acids and fatty acid methyl esters. *Biochim. Biophys. Acta* **863**, 115-120 (1986).

3. Chen P. Y., Pearce D. & Verkman A. S. Membrane water and solute permeability determined quantitatively by self-quenching of an entrapped fluorophore. *Biochemistry* **27**, 5713-5718 (1988).

4. Sacerdote M. G. & Szostak J.W. Semipermeable lipid bilayers exhibit diastereoselectivity favoring ribose. *Proc. Natl Acad. Sci. USA.* **102**, 6004-6008 (2005).
